# Cognition and epilepsy: Cognitive screening test

**DOI:** 10.1590/1980-57642020dn14-020013

**Published:** 2020

**Authors:** Glória Maria Almeida Souza Tedrus, Maria Lina Giacomino Almeida Passos, Letícia Muniz Vargas, Larissa Estela Ferreira Jacó Menezes

**Affiliations:** 1 Postgraduate Program in Health Sciences, Pontifical Catholic University of Campinas, Campinas, SP, Brazil.; 2 Undergraduate Student - Faculty of Medicine, Pontifical Catholic University of Campinas, Campinas, SP, Brazil.

**Keywords:** epilepsy, Brief Cognitive Battery-Edu, cognition, epilepsia, Brief Cognitive Battery-Edu, cognição

## Abstract

Cognitive deficits often occur in people with epilepsy (PWE). However, in Brazil, PWE might not undergo neurocognitive evaluation due to the low number of validated tests available and lack of multidisciplinary teams in general epilepsy outpatient clinics.

**Objective:**

To correlate Brief Cognitive Battery-Edu (BCB-Edu) scores with epilepsy characteristics of 371 PWE.

**Methods:**

Clinical and cognitive assessment (MMSE, BCB-Edu) of 371 PWE aged >18 years was performed. The clinical aspects of epilepsy were correlated with BCB-Edu data. Cognitive data of PWE were compared against those of 95 healthy individuals (NC), with p-<0.05.

**Results:**

People with epilepsy had lower cognitive performance than individuals in the NC group. Cognitive aspects also differed according to epilepsy characteristics. Predictive factors for impairment in multiple cognitive domains were age and use of more than one antiepileptic drug (logistic regression; R^2^ Nagelkerke=0.135).

**Conclusion:**

Worse cognitive performance was found in PWE on different domains. There was a relationship between cognitive impairment and the aspects of epilepsy. BCB-Edu proved to be effective as a cognitive assessment screening test for epilepsy in adults.

## RESUMO.

É frequente a ocorrência de déficits cognitivos em pessoas com epilepsia (PWE). Entretanto, no Brasil, as PWE podem ficar sem avaliação neurocognitiva pelo reduzido número de testes disponíveis e validados para nossa cultura e pela ausência de equipes multidisciplinares em ambulatórios gerais de epilepsia.

**Objetivo:**

Relacionar os escores do Brief Cognitive Battery-Edu (BCB-Edu) aos aspectos da epilepsia de 371 PWE.

**Métodos:**

Foi feita avaliação clínica e cognitiva (MMSE, BCB-Edu) de 371 PWE com idade > 18 anos. Foram relacionados os aspectos da epilepsia com os dados do BCB-Edu. Os dados cognitivos foram comparados aos de 95 indivíduos normais (NC), com p<0.05.

**Resultados:**

PWE apresentam desempenho cognitivo inferior ao do grupo NC. Os aspectos cognitivos foram distintos, segundo aspectos da epilepsia. Na regressão logística, os fatores preditivos para comprometimento em múltiplos domínios cognitivos foram a idade e a utilização de mais de uma droga antiepiléptica (R^2^ Nagelkerke=0.135).

**Conclusão:**

Houve, nas PWE, pior desempenho cognitivo em diferentes domínios. Houve relação entre déficit cognitivo com aspectos da epilepsia. O BCB-Edu mostrou-se eficaz como teste de triagem cognitiva na epilepsia em adultos.

Epilepsy can have a significant cognitive and behavioral impact and compromise social, academic and work performance, as well as quality of life, and increase stigma in people with epilepsy (PWE).^1^^,^ 2 Memory is negatively impacted by epilepsy, but this impairment may be more extensive and involve other cognitive functions.^2^^-^ 4 Epilepsy, in the recent classification by the International League Against Epilepsy (ILAE), is characterized as a brain network disease, while neuropsychological comorbidities have been included as an intrinsic part of the diagnosis of epilepsy.^5^

It is well known that, in Brazil, neurocognitive assessment is not often performed effectively in PWE cases, which are usually treated at general neurology outpatient clinics. This is due to several factors, such as the scarcity of adapted and validated tests for the Brazilian context, the scope of cognitive batteries and difficulties due to low educational levels, as well as the sociodemographic and cultural heterogeneity of the population, among others.^6^

The Brief Cognitive Battery-Edu (BCB-Edu) is well documented as a useful and accurate tool for cognitive screening in the assessment of individuals with different levels of formal education and those diagnosed with dementia.^7^^-^ 9 However, no studies using the BCB-Edu as a screening test in a general outpatient epilepsy clinic were found.

The hypothesis proposed by researchers involved in this study is that a significant number of PWE, despite reporting no complaints, present objective signs of cognitive dysfunction and are not adequately investigated. Cognitive screening can assess the basis of cognitive functioning, identify deficits and potentialities of individuals, and contribute to the analysis of the relationship between cognition and clinical aspects of epilepsy. Findings can help guide treatment planning and rehabilitation of potentially affected cognitive functions, thereby optimizing prognosis.

Thus, the objective of this study was to evaluate, in an epilepsy outpatient clinic, aspects of cognition using the BCB-Edu. Cognitive data were correlated with clinical and sociodemographic aspects and compared to those of a similar control group of healthy individuals (NC).

## METHODS

For this study, adult outpatients with epilepsy were recruited from the clinical neurology outpatient clinic of the *Pontifícia Universidade Católica de Campinas* (PUC-Campinas, Pontifical Catholic University of Campinas) Hospital in the city of Campinas, São Paulo, Brazil. For the recruitment, cases that met the following criteria were included: (1) a diagnosis of epilepsy according to the ILAE criteria;^5^ (2) individuals over 18 years old; (3) neurosurgically naive, and; (4) individuals who signed a consent term to undergo the procedures. The exclusion criteria were: (1) insufficient capacity to consent with, understand or answer the instruments; (2) the presence of other disabling chronic diseases.

In the control sample (NC), 95 healthy individuals with no history of neurological, psychiatric or other chronic disorders and a normal EEG, were recruited from the patients’ families in order to have similar sociodemographic data.

The Human Research Ethics Committee of the University approved the study under permit no. 73249517300005481. All participants signed an informed consent term.

The PWE who agreed to participate in the research underwent a clinical evaluation and research instruments were applied, with testing performed individually in a quiet, well-lit room at the neurological clinic of the university hospital. The assessment took place in a single session on the day patients attended the medical care unit at the hospital. All individuals followed the same neuropsychological and clinical research protocol.

The PWE and NC were submitted to the following procedures:

- A questionnaire on sociodemographic characteristics (age, gender, educational level, and hand dominance).
- All PWE underwent a neurological investigation involving the individual’s detailed medical record and collection of clinical epilepsy data (age at onset, seizure type and frequency, duration of epilepsy, and number of antiepileptic drugs (AED) taken), as well as the patient’s digital electroencephalogram (EEG) and MRI scans.
- Digital electroencephalogram (EEG): the location and side of epileptiform activity (EA) was evaluated.
- Mini-Mental State Examination (MMSE):^10^ This is a brief screening measure of cognition that assesses orientation, basic attention, working, learning, naming, construction, comprehension, and repetition memory. The maximum score is 30 points. The cut-off points for Brazil were established by Brucki et al.: 23 points for individuals with 1-4 years of formal education; 26 for individuals with 5-9 years; and 27 for individuals with 10 years or more;^11^
- Brief Cognitive Battery-Edu (BCB-Edu) 7: This was used to assess cognitive performance. It encompasses the identification of ten common pictures (naming) and immediate recollection (incidental recall). Subsequently, the pictures are presented again, and the subject is asked to memorize them for 30 seconds and then recall them (immediate recall). This procedure is repeated one more time (learning). Next, the subjects completed the semantic verbal fluency test (SVF) (animal pictures in one minute), the phonemic verbal fluency task (PVF) (oral fluency based on the letters F, A and S),^12^ and the Clock Drawing Test.^13^ After this, the subjects were asked to recall the pictures presented earlier (delayed recall). Finally, the same ten pictures were presented alongside ten distractor pictures, and the participant had to recognize the ones originally presented (recognition). For immediate memory, learning, delayed recall and recognition, the cut-off scores were <5, <7, <6 and <9, respectively.^7^ For the SVF test, a cut-off score of 9 was adopted for illiterate individuals and for subjects with <8 years of formal education; and of 13 for individuals with >8 years of formal schooling.^12^ The BCB-Edu was quick and easy to apply.

### Statistical analysis

The Statistical Packages for Social Sciences software, version 22, was used for the statistical analysis of this study. The statistical significance was set at a *p-*value < 0.05 in all tests.

The categorical variables were described using absolute values and percentages, while continuous variables were expressed as mean and standard deviations. Student’s *t*-test, analysis of variance (ANOVA) and the Pearson Chi-squared tests were used to compare the continuous and categorical variables. The Pearson correlation coefficient was used to measure the degree of associations among the quantitative variables.

The MMSE and the BCB-Edu scores of the PWE group were compared to those of the NC group, for similar ages, educational levels and sexes. Data for the PWE group cognitive were correlated with sociodemographic and clinical aspects of epilepsy. According to level of cognitive performance of PWE, the following groups were constructed: 1): individuals who had intact cognition and were minimally impaired when compared to NC; 2): individuals who had impaired SVF performance levels; and 3): individuals with impairment on multiple cognitive domains (memory, language and attention).

Based on the significant correlations, logistic regression tests were performed to calculate which clinical and sociodemographic aspects were associated with the poorest performers on the immediate memory test, learning test, delayed recall test, recognition test and the SVF. Logistic regression was also performed to analyze the PWE categorized as exhibiting impairment or no impairment on multiple cognitive domains. The relationship between the predictive variables and binary or continuous outcome variables (dependent variables) were studied, using variables with *p*<0.10 in the respective prior correlation analyses (independent variables). The following sociodemographic and clinical aspects were included in the equation: age; educational level; age at onset; seizure type (exclusively generalized, or focal); seizure frequency; duration of epilepsy; number of AEDs taken (1, or ≥2) and the type of epileptic syndrome (genetic, of unknown etiology, or structural).

## RESULTS

A total of 371 right-handed PWE (48.7% women) and 95 individuals from the NC (50.5% women) were evaluated. The PWE and control groups did not differ for gender, age or educational level.

According to the ILAE criteria, focal structural epilepsy was characterized in 228 (61.5%) cases; epilepsy of unknown etiology in 121 (32.6%) cases, and genetic epilepsy in 22 (5.9%) cases. Temporal lobe epilepsy with hippocampal sclerosis (TLE-HS), but not yet submitted to resective surgery, occurred in 113 cases (50 individuals with right TLE; 56 with left TLE; and 7 with bilateral TLE). At the time of this research, 238 (64.1%) individuals were taking one type of AED and 133 individuals were taking more than one AED. Seizures were exclusively generalized in 71 cases and focal in 300 cases. In cases of structural epilepsy (i.e. not TLE-HS), the identified etiology was: ischemic or hemorrhagic stroke, traumatic brain injury, cavernoma, among others.

The EA laterality on the EEG involved the right cerebral hemisphere in 126 cases and left hemisphere in 150 cases.

Sociodemographic and clinical aspects, according to syndrome, are shown in Table 1.

**Table 1:** Sociodemographic and clinical aspects of 371 people with epilepsy.

	Genetic	Focal unknown etiology	Focal structural	p
Sex: Female/ Male	13/9	58/63	110/190	0.607^a^
Age (years)	28.5 (±12.1)	47.2 (±16)	51.3 (±15.8)	<0.000^b^ *
Age at onset (years)	14 (±3.7)	26.1 (±19.7)	28.6 (±21.8)	0.039^b^ *
Antiepileptic drugs: 1/ ≥2	18/4	86/35	133/95	0.012^a^ *
Seizure frequency: with seizure / controlled	7/15	48/73	96/132	0.619^a^
EA laterality: right/ left	-	42/49	84/101	0.963^a^

EA: epileptiform activity;

a Chi-square test;

b Kruskall-Wallis;

* p<0.05.

Sociodemographic data and MMSE and BCB-Edu scores of PWE and NC are shown in Table 2. Cognitive performance on most tests was lower in the PWE group (n=371) when compared to the NC group.

**Table 2:** Sociodemographic aspects and Mini-Mental State Examination and Brief Cognitive Battery-Edu scores of people with epilepsy and controls.

		PWE (n=371)	NC (n=95)	P	TLE-HS (n=113)	NC (n=95)	p	Epileptic syndrome			
								Genetic	Unknown etiology	Structural	p
Age (years)		48.6 (±16.5)	48.5 (±10.4)	0.164^a^	48.3 (±13.4)	48.5 (±10.4)	0.881^a^	28.5 (±12.1)	47.2 (±16.0)	51.3 (±15.8)	<0.001* ^b^
Educational level (years)		5.7 (±3.8)	6.4 (±3.3)	0.074^a^	5.5 (±3.5)	6.4 (±3.3)	0.029* ^a^	8.6 (±3.4)	6.5 (±3.5)	5.0 (±3.8)	<0.001* ^b^
MMSE		23.1 (±4.1)	24.7 (±2.6)	<0.001* ^a^	22.8 (±4.5)	24.7 (±2.6)	<0.001* ^a^	25.1 (±3.8)	24 (±3.3)	22.5 (±4.4)	0.002* ^b^
BCB-Edu	• Identification	9.8 (±0.7)	9.9 (±0.2)	0.005* ^a^	9.9 (±0.2)	9.9 (±0.2)	0.723^a^	10.0 (±0.0)	9.8 (±0.7)	9.8 (±0.8)	0.370^b^
	- Naming	9.7 (±1.0)	9.8 (±0.5)	0.173^a^	9.8 (±0.3)	9.8 (±0.5)	0.423^a^	10.0 (±0.0)	9.8 (±0.7)	9.6 (±1.2)	0.094^b^
	• Incidental memory	5.8 (±1.8)	6.1 (±1.6)	0.086^a^	5.8 (±1.6)	6.1 (±1.6)	0.276^a^	6.4 (±1.8)	6.0 (±1.7)	5.6 (±1.8)	0.045* ^b^
	• Immediate memory	7.3 (±1.9)	8.2 (±1.4)	<0.001* ^a^	7.3 (±1.8)	8.2 (±1.4)	<0.001* ^a^	8.7 (±1.1)	7.5 (±1.8)	7.0 (±1.9)	<0.001* ^b^
	- Learning test	7.7 (±1.9)	8.9 (±1.1)	<0.001* ^a^	7.7 (±1.7)	8.9 (±1.1)	<0.001* ^a^	8.6 (±1.8)	8.2 (±1.7)	7.4 (±2.0)	<0.001* ^b^
	- Delayed recall	6.7 (±2.1)	8.1 (±1.5)	<0.001* ^a^	6.7 (±1.9)	8.1 (±1.5)	<0.001* ^a^	8.0 (±1.7)	7.0 (±2.0)	6.4 (±2.2)	0.003* ^b^
	- Recognition	9.1 (±1.6)	9.6 (±1.1)	<0.001* ^a^	9.3 (±1.2)	9.6 (±1.1)	0.037* ^a^	9.6 (±0.7)	9.2 (±1.4)	8.9 (±1.8)	0.031* ^b^
Clock Drawing Test		5.6 (±2.8)	6.9 (±2.5)	<0.001* ^a^	5.7 (±2.7)	6.9 (±2.5)	0.001* ^a^	7.3 (±2.6)	6.0 (±2.5)	5.1 (±2.9)	0.001* ^b^
SVF test		11.2 (±4.8)	12 (±4.8)	0.173^a^	10.7 (±4.3)	12 (±4.8)	0.045* ^a^	12.7 (±5.1)	12.3 (±4.8)	10.5 (±4.6)	0.001* ^b^
PVF (F)		4.9 (±3.9)	7.7 (±5.4)	<0.001* ^a^	4.4 (±3.0)	7.7 (±5.4)	<0.001* ^a^	5.6 (±3.6)	5.7 (±4.2)	4.5 (±3.7)	0.020* ^b^
PVF (A)		4.3 (±3.5)	7.0 (±4.7)	<0.001* ^a^	3.9 (±2.7)	7.0 (±4.7)	<0.001* ^a^	4.5 (±3.9)	5.2 (±3.7)	3.8 (±3.3)	0.003* ^b^
PVF (S)		4.2 (±3.5)	7.2 (±5.1)	<0.001* ^a^	3.8 (±2.6)	7.2 (±5.1)	<0.001* ^a^	5.1 (±3.9)	5.0 (±3.8)	3.8 (±3.1)	0.024* ^b^

PWE: people with epilepsy; NC: control group; TLE-HS: Temporal lobe epilepsy with hippocampal sclerosis; MMSE: Mini-Mental State Examination; BCB-Edu: Brief Cognitive Battery-Edu; SVF: semantic verbal fluency test; PVF: phonemic verbal fluency task.

a *t-*test;

b Kruskall-Wallis;

* *p*<0.05.

### Clinical and cognitive assessment

The individuals with focal structural epilepsy were older and had lower educational levels. Their cognitive performance regarding TLE-HS (n=113) score was worse when compared to the NC group. There was a significant difference in the MMSE and BCB-Edu scores according to type of epileptic syndrome (Table 2). There was no significant difference in the cognitive performance of patients with TLE-HS when compared to those with extratemporal epilepsy.

In 141 (38%) individuals, a decrease in the SVF test performance was observed. Also, a significantly lower performance was associated with the use of more than one AED. There was a deficit on the SVF test in patients with all epileptic syndromes, but with a significant difference (Chi-square; *p*=0.023). These deficits occurred in 9 (40.9%) individuals with genetic epilepsy; 34 (28%) individuals with focal epilepsy of unknown etiology; and in 98 (42.9%) individuals with focal structural epilepsy. There was no significant difference in SVF performance according to TLE-HS laterality.

When comparing cognitive aspects according to number of AEDs taken, PWE who used more than one AED had significantly lower performance levels on the MMSE (*t*-test; 22.4±4.1 vs 23.6±4.1; *p*=0.008); immediate memory (7.0±1.8 vs 7.5±1.9; *p*=0.017); delayed recall (6.5±2.3 vs 7.0±2.0; *p*=0.029); SVF (10.4±4.8 vs 11.7±4.8; *p*=0.011); and the PVF (word A) (3.7±3.2 vs 4.6±3.6; *p*=0.020) tests.

Analysis of cognitive performance data, revealed impairment of multiple domains in 238 (64.2%) individuals. One hundred and thirty-three individuals exhibited intact cognitive levels and were minimally impaired relative to individuals from the NC group. The association of cognitive impairment with sociodemographic and clinical aspects is shown in Table 3.

**Table 3:** Sociodemographic and clinical aspects according to occurrence of cognitive disorder.

		No cognitive impairment (n=133)	Impairment in multiple cognitive domains (n=238)	p-value
Age (years)		44.1 (±15.0)	51.1 (±16.9)	<0.001* ^a^
Educational level (years)		6.8 (±3.6)	5.0 (±3.7)	<0.001* ^a^
Age at onset (years)		23.2 (±15.9)	28.9 (±22.6)	0.005* ^a^
Duration of epilepsy (years)		20.9 (±15.3)	22.2 (±17.1)	0.459^a^
Sex	Female	76 (41.9%)	105 (58%)	0.016* ^b^
	Male	57 (30%)	133 (70%)	
Antiepileptic drugs	1	94 (70.6%)	39 (29.3%)	0.042* ^b^
	≥2	143 (60%)	95 (39.9%)	
Epileptic syndrome	Genetic	12 (54.5%)	10 (45.4%)	0.002* ^c^
	Focal unknown etiology	55 (45.4%)	66 (55.4%)	
	Focal structural	66 (28.9%)	162 (71%)	
Epilepsy	TLE-HS (113)	39 (34.5%)	74 (65.4%)	0.037* ^c^
	Unknown etiology and genetic	68 (47.5%)	75 (52.4%)	
TLE-HS laterality	Right (n=50)	20 (40%)	30 (60%)	0.261^b^
	Left (n=56)	18 (32.1%)	38 (67.8%)	

TLE-HS: Temporal lobe epilepsy with hippocampal sclerosis;

a *t*-test;

b Fisher's exact test;

c Chi-square test;

* *p*<0.05.

The values for correlation ​​of MMSE and BCB-Edu scores with age, age at onset and duration of epilepsy are shown in Table 4.

**Table 4:** Correlation of Mini-Mental State Examination and Brief Cognitive Battery-Edu scores with age, age at onset and duration of epilepsy.

		Age			Age at onset			Duration of epilepsy	
		Correlation	p-value		Correlation	p-value		Correlation	p-value
MMSE		-0.190	<0.001*		-0.012	0.822		-0.174	0.001*
BCB-Edu	Identification	-0.155	0.003*		-0.098	0.061		-0.032	0.542
	Naming	-0.170	0.001*		-0.122	0.019*		-0.017	0.743
	Incidental memory	-0.388	<0.001*		-0.291	<0.001*		-0.029	0.573
	Immediate memory	-0.345	<0.001*		-0.185	<0.001*		-0.115	0.028*
	Learning test	-0.308	<0.001*		-0.218	<0.001*		-0.035	0.506
	Delayed recall	-0.231	<0.001*		-0.190	<0.001*		0.004	0.943
	Recognition	-0.308	<0.001*		-0.223	<0.001*		-0.031	0.548
Clock Drawing Test		-0.220	<0.001*		-0.069	0.200		-0.133	0.013*
SVF test		-0.180	<0.001*		-0.099	0.058		-0.060	0.254
PVF (F)		-0.119	0.023*		-0.002	0.965		-0.118	0.025*
PVF (A)		-0.064	0.223		-0.007	0.894		-0.058	0.273
PVF (S)		-0.101	0.053		-0.016	0.756		-0.082	0.121

MMSE: Mini-Mental State Examination; BCB-Edu: Brief Cognitive Battery-Edu; SVF: semantic verbal fluency test; PVF: phonemic verbal fluency task; Pearson correlation;

* *p*<0.05.

There was no difference in cognitive performance according to frequency and type of seizures or EA laterality on the EEG.

In the 371 PWE, when assessing which sociodemographic aspects were associated with lowest performers on the immediate memory, learning and recognition tests, the logistic regression test revealed that the variables retained in the equation were individuals with the lowest educational level and highest age bracket (Table 5).

**Table 5:** Odds ratio for factors associated with poorer cognitive performance in 371 PWE.

Test	Variables in equation	Beta coefficient	SE	95% CI for coefficient	p
Immediate memory^a^	Age	0.035	0.011	1.015 1.057	0.001*
	Educational level (years)	-0.091	0.046	0.825 0.999	0.047*
Learning test^b^	Age	0.027	0.010	1.008 1.047	0.005*
	Educational level (years)	-0.104	0. 042	0.830 0.978	0.013*
Delayed recall^c^	Educational level (years)	-0.114	0.036	0.831 0.958	0.002*
	Age at onset	0.015	0.006	1.003 1.027	0.017*
	AED: 1/ ≥2	-0.785	0.268	0.270 0.771	0.003*
Recognition^d^	Age	0.030	0.010	1.011 1.052	0.003*
	Educational level (years)	-0.102	0.045	0.827 0.986	0.022*
SVF test^e^	Educational level (years)	-0.186	0.032	0.780 0.884	<0.001*
	AED: 1/ ≥2	-0.740	0.255	0.289 0.787	0.004*
Impairment in multiple cognitive domains ^f^	Age	0.026	0.006	1.007 1.046	0.006*
	AED: 1/ ≥2	-0.628	0.285	0.035 0.934	0.028*

AED: number of antiepileptic drugs taken; SVF: semantic verbal fluency test; SE: standard error; CI:

a R^2^ Nagelkerke=0.130;

b R^2^ Nagelkerke=0.119;

c R^2^ Nagelkerke=0.109;

d R^2^ Nagelkerke=0.124;

e R^2^ Nagelkerke=0.169;

f R^2^ Nagelkerke =0.072.

In the logistic regression test used to determine which aspects were correlated with lowest performance on the delayed recall task, significant associations were observed with age at onset of epilepsy, use of >1 AED and lower educational level.

On logistic regression, lower performance on the SVF test was correlated with the use of >1 AED and lower educational level (Table 5).

The clinical aspects age and use of >1 AED were associated with impairment of multiple cognitive domains on logistic regression. The other clinical aspects were excluded from the equation. The effect size was small and medium for this model (Table 5).

## DISCUSSION

In this study, results showed that patients with different types of epilepsy performed worse on neuropsychological testing across almost all domains of cognitive function when compared with a similar individual from the NC group. These findings are consistent with the results of previous studies.^1^^,^ 2 ^,^ 14 In the literature, most studies evaluate PWE in relation to refractory and focal seizures, and candidates for surgery, with an extensive neuropsychological battery of exams for preoperative evaluation in tertiary and postoperative services and in centers specializing in epilepsy surgery.^3^^,^ 6

In this sample, some level of impairment was observed in several domains of cognitive functioning, with lower performance on naming, memory (immediate memory, learning, delayed recall, recognition) and clock drawing tests, on the PVF and for global MMSE score. In epilepsies, cognitive impairment tends to be heterogeneous, as do the types of epilepsies; with substantial variability and differences in performance, ranging from significant impairment to intact cognitive functions.^1^^,^ 2 There is a rich body of literature describing various factors in the course of the disease that may contribute to cognitive impairment, including etiology, topography of the epileptiform area, persistent interictal abnormalities on EEG, pathogenetic mechanisms, the cumulative factor of seizures, presence of psychiatric comorbidities, among others.^14^^-^ 22 More recent studies addressing TLE-HS have proposed the term “cognitive phenotypes” for the characterization of subgroups of this epilepsy with distinct profiles of cognitive involvement.^4^^,^ 23 It is believed the study of cognitive phenotypes and correlations with clinical, neuroanatomical and psychosocial aspects may contribute to the planning of specific treatments.^4^^,^ 23

Approximately one third of the present sample exhibited intact cognitive functions and were minimally impaired compared to the individuals in the NC group, a finding consistent with data from other studies.^4^^,^ 14 ^,^ 16 ^,^ 23 These PWE were significantly younger, have shorter disease duration and higher educational levels, female, made use of only one AED, and had epilepsy of unknown etiology and genetic epilepsy.

As expected, lower educational level (inadequate schooling) was a predictor of worse performance on the cognitive assessments of PWE. Higher educational level and higher mental activity appear to be protective factors for cognition by increasing cognitive-related neural networks.

### Cognitive performance and clinical aspects of epilepsy

A poorer performance in memory, attention and language was correlated with disease duration, patient age and earlier onset of epilepsy, similar to findings of other authors.^16^^,^ 24 ^-^ 26 However, which pathophysiological mechanisms are involved remain unclear. Early-onset epilepsies with seizures, and those with prolonged duration, tend to cause greater brain insult and consequent vulnerability of cognitive functions.^27^^,^ 28 On the other hand, some chronic epileptic syndromes may, in specific situations, induce brain plasticity in eloquent areas, thus leading to a process of neuronal functional reorganization of intrahemispheric or interhemispheric cortical adjacent areas, which may provide for the maintenance of cognitive functions.^2^^,^ 15 ^,^ 29

Longitudinal studies evaluating cognitive aspects in chronic epilepsy of adults who have not been submitted to epilepsy surgery are scarce. It seems that the cognitive functions remain relatively stable for the first 5-10 years of the disease, while cognitive impairment progresses slowly over ensuing decades without the characteristics of a dementia-like progressive disease; some studies suggest that in a number of successfully treated cases, these individuals have a good cognitive prognosis.^1^^,^ 19 ^,^ 30 ^,^ 31 However, it is still controversial as to when cognitive impairment begins in the course of the disease, or whether this impairment is present before the onset of epilepsy and whether cognitive deficits are caused by brain diseases underlying epilepsy.^4^^,^ 14 ^,^ 32

Cognitive performance did not differ significantly between TLE-HS and extratemporal epilepsy cases, contrasting with other studies.^20^^,^ 26

The use of more than one AED was associated with poorer performance in memory (immediate and delayed recall) and on fluency tests (category and phonemic), as well as with lower total MMSE score. where multiple cognitive domains were involved in these cases. AEDs may negatively impact cognitive functions.^24^^,^ 25 ^,^ 33 The use of more than one AED is associated with increased risk of cognitive decline, particularly in executive and attention functions and, to a lesser extent, of memory impairment.^33^ Other studies report that the side effects of AEDs seem to be reversible after their discontinuation and experimental studies have shown a neuroprotective effect of AEDs, albeit with unknown clinical significance.^24^^,^ 25 ^,^ 33

Verbal fluency (VF), which evaluates lexical recall ability, semantic knowledge and mental flexibility, is compromised in many cases and in different epileptic syndromes. In the literature, most studies evaluate VF in frontal and temporal lobe epilepsies.^34^ Similarly to several other studies, the researchers observed SVF impairment in the TLE-HS (right and left) group when compared to the NC group.^32^^,^ 35 However, no significant difference was found in VF performance according to TLE-HS laterality. Verbal fluency deficits are often interpreted as a sign of left hemisphere dysfunction.^35^

The involvement of several cognitive domains besides memory was more evident in cases with temporal lobe impairment (TLE-HS) compared to PWE presenting normal EEG images (focal epilepsy of unknown etiology) or compared to the NC group, mirroring results found in the literature.^4^^,^ 15 ^,^ 16 The cognitive dysfunctions observed in focal epilepsies, particularly in TLE-HS, extend beyond the epileptogenic zone, lobe or hemisphere and involve extra-temporal regions, subcortical structures and cerebellum. This suggests that cognitive impairment in TLE-HS is not linked to specific structure functions, but goes beyond the epileptogenic focus, affecting distinct cognitive abilities, suggesting network disruption^4^^,^ 23 ^,^ 24 and corresponding failure of network flexibility.^36^^,^ 37

This study has some limitations. Although the study used a standardized, scientifically validated instrument, its researchers believe that there are certain limitations in as far as the sample involved a single institution, precluding a cross-cultural comparison. It was not possible to compare the findings of this study using the BCB-edu with data from other studies to assess cognition in epilepsy. Another potential limitation is that the results of the cognitive assessments were not compared to those of other validated neuropsychological tests in Brazil.

In conclusion, lower cognitive performance was observed in PWE, as measured by the BCB-Edu. There was a relationship between cognitive impairment and aspects of epilepsy. The data suggest the need for inclusion of cognitive tracking assessment in the care of PWE within general outpatient clinics, as this would provide clinical support for medical care and treatment planning and, if necessary, support indication for cognitive rehabilitation.

Author contributions. All of the above authors were involved in data collection and writing the article. Glória Tedrus was responsible for preparing the project, providing medical care to patients and for the ethical aspects involved in the research.
